# The synthesis and characterization of Cu_2_ZnSnS_4_ thin films from melt reactions using xanthate precursors

**DOI:** 10.1007/s10853-017-1367-0

**Published:** 2017-07-20

**Authors:** Mundher Al-Shakban, Peter D. Matthews, Nicky Savjani, Xiang L. Zhong, Yuekun Wang, Mohamed Missous, Paul O’Brien

**Affiliations:** 1 School of Materials, University of Manchester, Oxford Road, Manchester, M13 9PL UK; 2 School of Chemistry, University of Manchester, Oxford Road, Manchester, M13 9PL UK; 3 School of Electrical and Electronic Engineering, University of Manchester, Oxford Road, Manchester, M13 9PL UK

## Abstract

Kesterite, Cu_2_ZnSnS_4_ (CZTS), is a promising absorber layer for use in photovoltaic cells. We report the use of copper, zinc and tin xanthates in melt reactions to produce Cu_2_ZnSnS_4_ (CZTS) thin films. The phase of the as-produced CZTS is dependent on decomposition temperature. X-ray diffraction patterns and Raman spectra show that films annealed between 375 and 475 °C are tetragonal, while at temperatures <375 °C hexagonal material was obtained. The electrical parameters of the CZTS films have also been determined. The conduction of all films was p-type, while the other parameters differ for the hexagonal and tetragonal materials: resistivity (27.1 vs 1.23 Ω cm), carrier concentration (2.65 × 10^+15^ vs 4.55 × 10^+17^ cm^−3^) and mobility (87.1 vs 11.1 cm^2^ V^−1^ s^−1^). The Hall coefficients were 2.36 × 10^3^ versus 13.7 cm^3^ C^−1^.

**Electronic supplementary material:**

The online version of this article (doi:10.1007/s10853-017-1367-0) contains supplementary material, which is available to authorized users.

## Introduction

Copper zinc tin sulfide (Cu_2_ZnSnS_4_, CZTS) is a promising absorber layer for use in photovoltaic cells and is composed of low toxicity [1–3] and earth-abundant elements [4–6]. It has a large absorption coefficient (*α* ≥ 10^4^ cm^−1^) and a direct band gap of about 1.45 eV [7–10]. The performance of CZTS absorber layers is still less than that of silicon [11], which is the current industrial standard; however, unlike silicon, it has the advantage of a direct and tunable band gap [12].

CZTS thin films have been prepared using several techniques, including: chemical vapor deposition (CVD) [13], co-sputtering followed by sulfurization from the vapor phase [14], chemical bath deposition (CBD) [15], successive ionic layer adsorption and reaction (SILAR) [16] and solvothermal treatment of a layered elemental (copper, zinc and tin) film with sulfur powder [17]. However, there are two major problems with routes that involve the deposition of the individual components (either as metal M or M_*x*_S_*y*_) followed by high-temperature annealing in a sulfur atmosphere. The first issue is the loss of volatile components such as SnS. The photoelectrical performance of CZTS is highly dependent on good stoichiometric control, and such evaporation can make it difficult to control the composition of the target phase [18–22]. The second issue is the stability of the Mo electrode that the CZTS is often deposited on; this can react with sulfur to form a MoS_2_ layer between the electrode and the CZTS, resulting in a dramatic decrease in performance of the photovoltaic device [23, 24]. These two problems indicate a requirement for a new synthetic route to CZTS that avoids higher temperatures.

The Cu_2_ZnSnS_4_ unit cell is based on zinc blende: It is related to the chalcopyrite structure of CuInS_2_ by changing indium for zinc and tin, with the metals in tetrahedral coordination [25]. There are three different phases of CZTS: kesterite, stannite and primitive mixed CuAu-like structure (PMCA) [26–28]. Zunger et al. [29, 30] have established that strain energy is responsible for the stability of the CZTS crystal structure. They found that for large lattice mismatches, the kesterite structure has a lower strain energy, which means that chalcopyrite is more stable than the CuAu-like structure; as a result, the stability of kesterite is higher than that of stannite and PMCA, but there is little difference in strain energy between the kesterite and stannite structures (~3 meV/atom).

Walsh et al. have performed calculations on the total energy of CZTS and other quaternary semiconductors of the form I_2_-II-IV-VI_4_ (I=Cu, Ag; II=Zn, Cd; IV=Si, Ge, Sn; VI=S, Se). They found that the kesterite structure is thermodynamically more stable than stannite, wurtzite-kesterite and wurtzite-stannite [31–33]. In stannite, (Cu/Fe)Sn layers alternate with Cu_2_ layers in the unit cell, whereas for kesterite CuSn alternates with CuZn. The similar lattice constants and total energy values for kesterite and stannite CZTS mean that there is often the possibility of both phases existing together depending on the methods used to prepare the material [34].

A big disadvantage of CZTS in photovoltaic applications is its ability to crystallize in these different forms. Schorr et al. [35] synthesized tetragonal CZTS at 860 °C and the cubic phase of CZTS at temperatures >885 °C by the solid-state reaction of the pure elements in sealed evacuated silica tubes. Brandl et al. [36] synthesized CZTS nanoparticles in a disordered cubic structure by the hot injection of CuCl_2_, Zn(OAc)_2_ and SnCl_2_ with S in oleylamine at 225 °C, and they found that a disordered cubic phase changed to the tetragonal CZTS phase at 275 °C. Cattley et al. [37] have also observed a temperature-dependent phase change from tetragonal kesterite to hexagonal wurtzite during their synthesis of quantum dots from Cu(acetylacetonate)_2_, Zn(OAc)_2_, SnCl_4_ and S(SiMe_3_)_2_. Nakayama et al. [38] first successfully prepared stannite CZTS thin film via spray pyrolysis, and its electronic properties have been theoretically assessed by Marques et al. [39] and Scarpulla et al. [40]. The development of a method for controlling the phase of the obtained CZTS would be a major step toward commercializing CZTS. We have previously reported the synthesis of CZTS and Cu_2_Zn_1−x_Fe_x_SnS_4_ nanoparticles and thin films from the decomposition of dithiocarbamate single-source precursors (SSPs) [41–43].

Recently xanthates have been used as SSPs to metal sulfide nanocrystals [44–49]. They have the general chemical formula [M(S_2_COR)_n_], where R is an alkyl group. Xanthates are good precursors to deposit metal sulfide thin films as the preformed M–S bonds make the conversion to a metal sulfide film straightforward [50, 51]. They can also decompose at lower temperatures compared to other precursors, and they are usually held to decompose by the Chugaev elimination reaction [52]. A range of xanthates and a parallel range of dithiocarbamates have been synthesized by Molloy and co-workers as sources of metal sulfides. They studied thermal decomposition profiles by thermogravimetric analysis (TGA), and their experiments show that metal xanthates are viable precursors for Cu_2_ZnSnS_4_, in both thin film and nanoparticulate form [53].

In this report, we discuss the synthesis of a range of copper, zinc and tin *O*-ethyl and *O*-*n*-butylxanthates and assessed their suitability as precursors to CZTS. On the basis of decomposition properties [(Ph_3_P)_2_CuS_2_COEt], [Zn(S_2_CO^n^Bu)_2_] and [Sn(S_2_COEt)_2_] have been used as coating precursors for the production of Cu_2_ZnSnS_4_ films on glass. We focus on both the annealing temperature and the role of the xanthate ligand in the decomposition process for the potential in control of the structural and electronic properties of the CZTS films produced.

The CZTS films were analyzed by powder X-ray diffraction (p-XRD), Raman spectroscopy, scanning electron microscopy (SEM) and transmission electron microscopy (TEM). We investigated the electrical properties using the van der Pauw method [54], and the resistivity of the films was calculated from the Hall voltage and sheet resistance.

## Experimental

All chemicals, with the exception of tin(II) chloride (Alfa Aesar), were purchased from Sigma-Aldrich and were used as received. Elemental analysis (EA) and thermogravimetric analysis (TGA) were carried out by the Microelemental Analysis service at the University of Manchester. EA was performed using a Flash 2000 Thermo Scientific elemental analyzer, and TGA data were obtained with Mettler-Toledo TGA/DSC1 star^e^ system between the range of 30–600 °C at a heating rate of 10 °C min^−1^ under nitrogen flow. Scanning electron microscopy (SEM) images were obtained using a Philips XL30 FEG, with energy-dispersive X-ray spectroscopy (EDX) data obtained using a DX4 instrument. Samples suitable for transmission electron microscopy (TEM) were prepared by exfoliating thin films in toluene and dropcasting the suspension onto holey carbon support grids, which were then air-dried. TEM was performed using Philips CM20 equipped with a LaB_6_ source (Fig. 5a, b) or a FEI Tecnai G2 F30 with Schottky field emitter operated at 300 keV (Fig. 5c). Powder X-ray diffraction (p-XRD) analyses were carried out using an X’pert diffractometer with a Cu–K_*α*1_ source (*λ* = 1.54059 Å), the samples were scanned between 20 and 75°, and the applied voltage was 40 kV and the current 30 mA. Raman spectra were measured using a Renishaw 1000 Micro-Raman System equipped with a 514 nm laser.

### Synthesis of metal xanthate complexes

#### Synthesis of potassium *n*-butylxanthate ligand

The synthesis of [K(S_2_CO^n^Bu)] was adapted from a literature procedure [55]. KOH (5.64 g, 0.10 mol) and ^n^BuOH (50 ml) were stirred for 2 h at room temperature, and then, CS_2_ (7.73 g, 6.11 ml, 0.10 mol) was added dropwise to the reaction, resulting in an orange solution. The unreacted alcohol was removed *in vacuo,* and the yellow solid product was dried and recrystallized from *n*-butyl alcohol to give [K(S_2_CO^n^Bu)] (13.45 g, 71.5 mmol, 71.5% yield). MPt: 232–235 °C.

Calc. for C_5_H_9_KOS_2_ (%): C 31.9, H 4.82, S 34.0, K 21.8; found: C 31.6, H 4.51, S 33.3, K 22.0.

FT-IR (cm^−1^): 2958 (m), 2869 (w), 1461 (m). 1445 (w), 1261 (s), 1149 (m), 1173 (m), 1062 (m), 1014 (m), 747.3 (m), 669.0 (s), 566.2 (s).

#### Synthesis of bis(*O*-butylxanthato)zinc(II)

The complex [Zn(S_2_CO^n^Bu)_2_] was synthesized by a similar method to [Zn(S_2_COEt)_2_] using [K(S_2_CO^n^Bu)] (5.00 g, 0.027 mol) and ZnCl_2_ (1.81 g, 0.013 mol). Yield = 4.61 g, 0.013 mol, 97%. MPt: 105–112 °C.

Calc. for C_10_H_18_O_2_S_4_Zn (%): C 33.0, H 4.99, S 35.2, Zn, 18.0; found: C 33.0, H 4.99, S 35.2, Zn 18.0.

FT-IR (cm^−1^): 2952 (w), 2868 (w), 1463 (w). 1189 (s), 1129 (m), 1040 (s), 939.3 (w), 736.3 (w), 665.1 (w).

#### Synthesis of bis(*O*-ethylxanthato)tin(II)

[Sn(S_2_COEt)_2_] was prepared by a procedure modified from that described in the literature [53, 56]. An aqueous solution of potassium ethylxanthate (10.0 g, 0.062 mol) was added to a solution of tin(II) chloride (5.90 g, 0.031 mol) in distilled water (100 ml) and stirred for a further 30 min. The yellow precipitate produced was collected by vacuum filtration, washed with water (3 × 50 ml) and finally dried in a vacuum oven at room temperature for 2 h to give [Sn(S_2_COEt)_2_] (7.20 g, 0.020 mol, 64% yield). MPt: 46–49 °C.

Calc. for C_6_H_10_O_2_S_4_Sn (%): C 20.0, H 2.79, S 35.5, Sn 32.9; found: C 19.7, H 2.74, S 35.5, Sn 32.2.

FT-IR (cm^−1^): 2986 (w), 2930 (w), 1457 (w), 1355 (w), 1196 (s), 1108 (s), 1021 (s), 852.0 (w), 801.3 (w), 563.4 (w).

#### Synthesis of (*O*-ethylxanthato)copper(I) triphenylphosphine

A solution of potassium ethylxanthate (0.641 g, 0.0040 mol) in chloroform (40 ml) was added to a solution of triphenylphosphine (2.09 g, 0.008 mol) and CuCl (0.40 g, 0.0040 mol) in the same amount of chloroform. A white precipitate was obtained after continuous stirring for 1 h at room temperature. The solution was filtered to obtain a clear yellow solution. Cooling the yellow solution to −20 °C gave yellow crystals of *O*-ethylxanthato copper(I) triphenylphosphine (2.40 g, 0.0033 mol, 85% yield). MPt: 185–191 °C.

Calc. for C_39_H_35_CuOP_2_S_2_ (%): C 66.1, H 4.97, S 9.02, P 8.74, Cu 8.96; found: C 65.7, H 5.08, S 8.77, P 8.44, Cu 8.74.

FT-IR (cm^−1^): 3048 (w), 2992 (w), 1478 (m) 1433 (m), 1290 (s), 1142 (m), 1041 (m), 1009 (s), 849.5 (s), 740.8 (m), 617.7 (s), 559.2 (s).

Additional precursors that were not used to make thin films are described in ESI.

### Preparation of thin films

Glass slides were cut to 20 mm × 15 mm, cleaned by sonication in acetone and water and allowed to dry. Coating solutions were prepared by dissolving [(Ph_3_P)_2_CuS_2_COEt] (0.82 mmol), [Zn(S_2_CO^n^Bu)_2_] (0.41 mmol) and [Sn(S_2_COEt)_2_] (0.41 mmol) in THF (6 ml). A clear yellow solution was obtained. For each sample 2 ml of solution was coated onto the glass slide by spin coating at 700 rpm for 120 s and allowed to dry. The resulting films were then annealed in an N_2_ atmosphere with a heating ramp ~3 °C min^−1^ and held at the target temperature for 120 min; after this time had elapsed, the furnace was turned off and the tube allowed to cool to room temperature. The films were kept in the N_2_ atmosphere until they had cooled to room temperature.

### Electrical measurements

The electrical properties of the thin films were investigated using the van der Pauw method [54]; contacts to the 7.5 × 7.5 mm CZTS thin films were obtained using pure In probes. A magnetic field of 0.088T strength was applied during the Hall measurements. The values of resistance between each In contact pairs were homogeneous, and the four individual Hall voltages were close to each in value giving a statistically meaningful average. The film resistivity was calculated from the Hall voltage and sheet resistance.

## Results and discussion

A range of copper, zinc and tin ethyl- and *n*-butylxanthate complexes were synthesized by the reaction of the appropriate metal chloride with the relevant potassium xanthate. The suitability of these complexes for melt reactions was assessed through measurements of their thermal stability.

**Figure 1:**
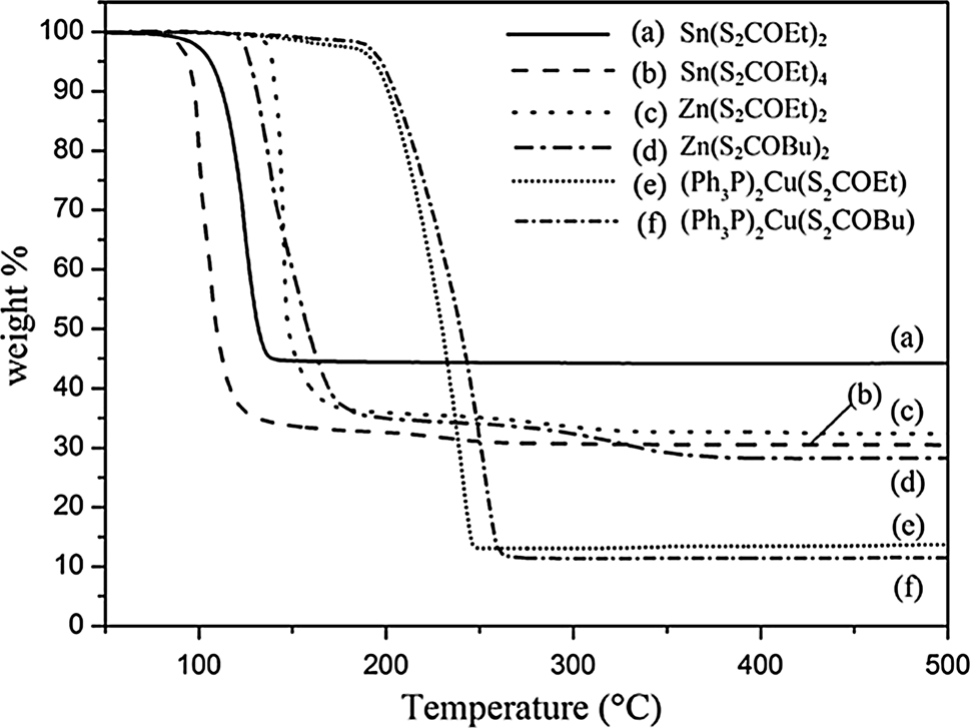
Decomposition profiles of the six potential CZTS precursors that were assessed by thermogravimetric analysis (TGA). Heating rate of 10 °C min^−1^ under N_2_ flow

Thermogravimetric analysis (TGA, Fig. 1) demonstrates the decomposition range of the Sn, Zn and Cu xanthates in a nitrogen atmosphere. An optimum mixture of precursors for melt reactions is one in which the precursors all decompose at similar reaction temperatures.

[Sn(S_2_COEt)_2_] decomposes cleanly in TGA at 100–145 °C, whereas [Zn(S_2_CO^n^Bu)_2_] decomposes via a two-step process between 110–220 and 250–350 °C. The decomposition of [(Ph_3_P)_2_CuS_2_COEt] shows a mass loss starting at about 140 °C and finishing at 260 °C. The TGA curves indicate that SnS and CuS are formed cleanly, while ZnS contains impurities up to 350 °C. Sn(S_2_COEt)_4_ and (Ph_3_P)_2_(Cu(S_2_CO^n^Bu)_2_ have hence been discounted as viable precursors owing to their decomposition temperatures that differ from other precursors. The ethyl xanthate of zinc [Zn(S_2_COEt)_2_] is much more hydroscopic than its *n*-butyl cousin. Therefore, [(Ph_3_P)_2_CuS_2_COEt], [Zn(S_2_CO^n^Bu)_2_] and [Sn(S_2_COEt)_2_] were chosen to deposit CZTS films. Additionally, these complexes are readily soluble in many common organic solvents.

**Figure 2:**
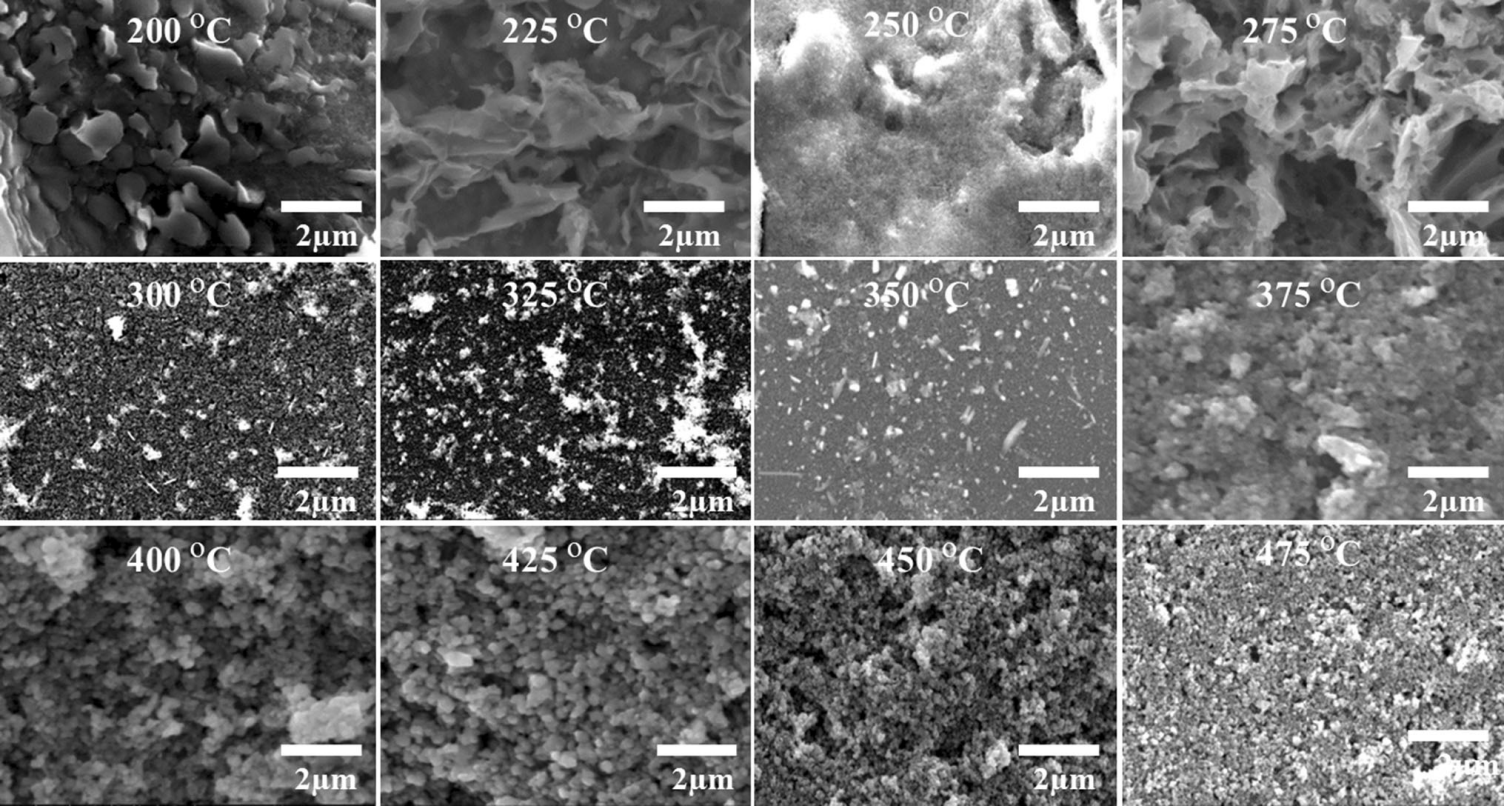
SEM images showing the surface morphology of the CZTS films obtained by heating spin-coated films at various temperatures

CZTS thin films were prepared using a solution of [(Ph_3_P)_2_CuS_2_COEt] (0.82 mmol), [Zn(S_2_CO^n^Bu)_2_] (0.41 mmol) and [Sn(S_2_COEt)_2_] (0.41 mmol) dissolved in THF (6 ml) and spin coated onto a glass slide. The resulting films were then heated in an N_2_ atmosphere at the desired temperature (between 200 and 475 °C) for 120 min. The CZTS films were gray for all of the heating temperatures. Scanning electron microscopy (SEM) images (Fig. 2) reveal the surface morphology of the films to be predominantly granular for the films prepared at temperatures >300 °C, with particles in the region of 0.25 μm in diameter. The films prepared at lower temperature have a more flake-like consistency and contain much smaller particles. Side-on SEM images (ESI Figure S5) show that the films are 1.85–2.0 μm in thickness.

Energy-dispersive X-ray spectroscopy (EDX) analysis shows the presence of copper, zinc, tin and sulfur in the films. It is important to determine the Zn/Sn and Cu/(Zn + Sn) ratios within the films as slight changes in these values can lead to significant changes in structural/electronic properties [57]. The EDX measured compositions of the as-prepared films are shown in ESI Table S1.

The Cu/(Zn + Sn) ratio is in the range of 0.8–1.0 (ESI Table S1). The films heated at 225 °C gave Cu/(Zn + Sn) > 1, whereas the other films heated at high temperatures are copper deficient (ESI Table S1), which may reflect the volatility of the precursors. The slight copper deficiency is a promising result as solar cells made from Cu-poor films perform substantially better than those made from stoichiometric Cu_2_ZnSnS_4_ [18].

**Figure 3:**
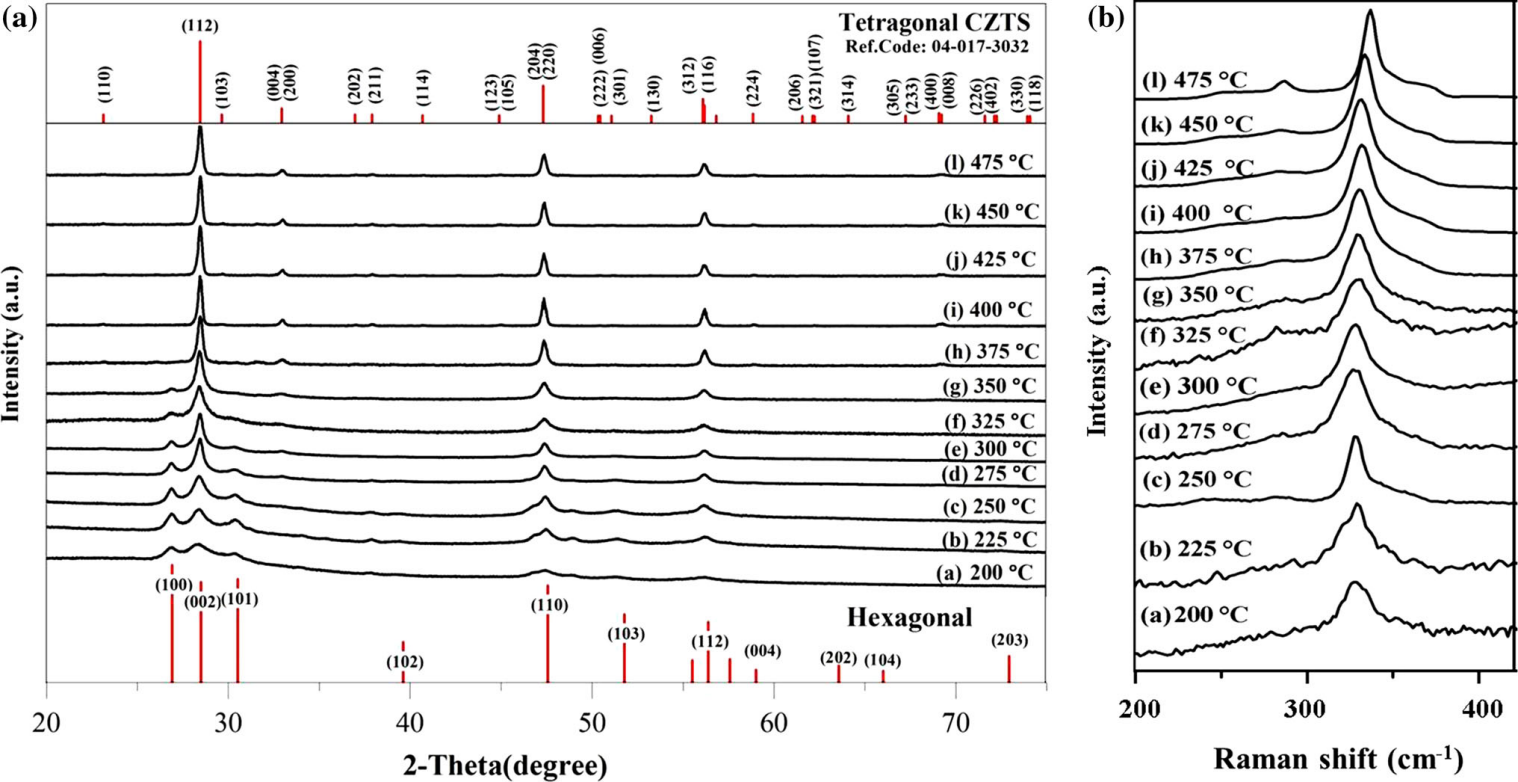
**a** p-XRD patterns and **b** Raman scattering spectra of CZTS thin films annealed in an N_2_ atmosphere at the desired temperature (200–475 °C) for 120 min. Chalcocite (Cu_2−x_S) is identifiable within the p-XRD for temperatures <300 °C: low-intensity peaks for the (110) plane (2*θ* = 46.7°) and (103) plane (2*θ* = 48.8°)

The p-XRD patterns for the samples prepared at temperatures between 200 and 475 °C are shown in Fig. 3. It is clear that the peaks sharpen with increased heating temperatures, with the width of the (002)/(112) reflection decreasing, which indicates improved crystallinity or increased crystalline size.

At temperatures ≥375 °C simpler diffraction patterns are seen than at lower temperatures. Only KS-CZTS was observed—this grows with a strongly preferred (112) orientation and matches well with the tetragonal CZTS standard (JCPDS No. 04-017-3032) which has a space group Ī4. The lattice parameters of a = 5.431 Å, b = 5.431 Å and c = 10.844 Å (ESI Tables S4, S5 and S6) match well with the literature values and indicate that the material is tetragonal and not cubic [58].

The films prepared at 200–250 °C consist of a relatively poorly crystalline hexagonal CZTS (WZ-CZTS, ESI Tables S2 and S6). The wurtzitic form of CZTS has previously been seen for nanoparticles and thin films [37, 59]. The diffraction patterns of the hexagonal and orthorhombic phases are very similar. However, in orthorhombic CZTS the low angle peaks (100) and (101) in Fig. 3a appear as ‘doublets’, representing the (210) + (020) and (211) + (021) lattice planes, respectively [60]. This splitting is not observed in our p-XRD pattern, nor is it seen in the selected area electron diffraction (SAED, Fig. 5a and ESI Table S2); indeed, the patterns from TEM are very sharp and clear. Therefore, we conclude that the low-temperature films result in the WZ-CZTS phase. Some contamination with binary or ternary phases may occur, as can be observed in the films prepared at <300 °C in which chalcocite (Cu_2−x_S) is identifiable within the diffraction patterns.

The Raman spectra for the CZTS films give a good indication of the phase formed (Fig. 3b). Kesteritic CZTS has a relatively narrow and dominant Raman shift at 338 cm^−1^ from the A_1_ mode, with further peaks at 288 (A mode), 358 (B mode) and 372 cm^−1^ (B mode) [61, 62]. This is seen in the films heated at higher temperatures (>400 °C). For the samples prepared at lower temperature (<300 °C), the major peak has a shift of 327 cm^−1^ (ESI Figure S3). This corresponds to the WZ-CZTS A mode [63]. This indicates that the low-temperature films are wurtzite CZTS and there is a phase transition to kesterite at higher temperatures, in agreement with the p-XRD data. We note that there is also a peak in the Raman spectra for the 200 °C film at 468 cm^−1^, which corresponds to Cu_2_S.

Increasing in temperature leads to a gradual narrowing of the bands, and Raman bands appear with frequencies of about 331, 333 and 337 cm^−1^. Films heated at 475 °C showed a higher intensity band at 337 cm^−1^ (ESI Figure S4), which is indicative of KS–CZTS [64].

**Figure 4:**
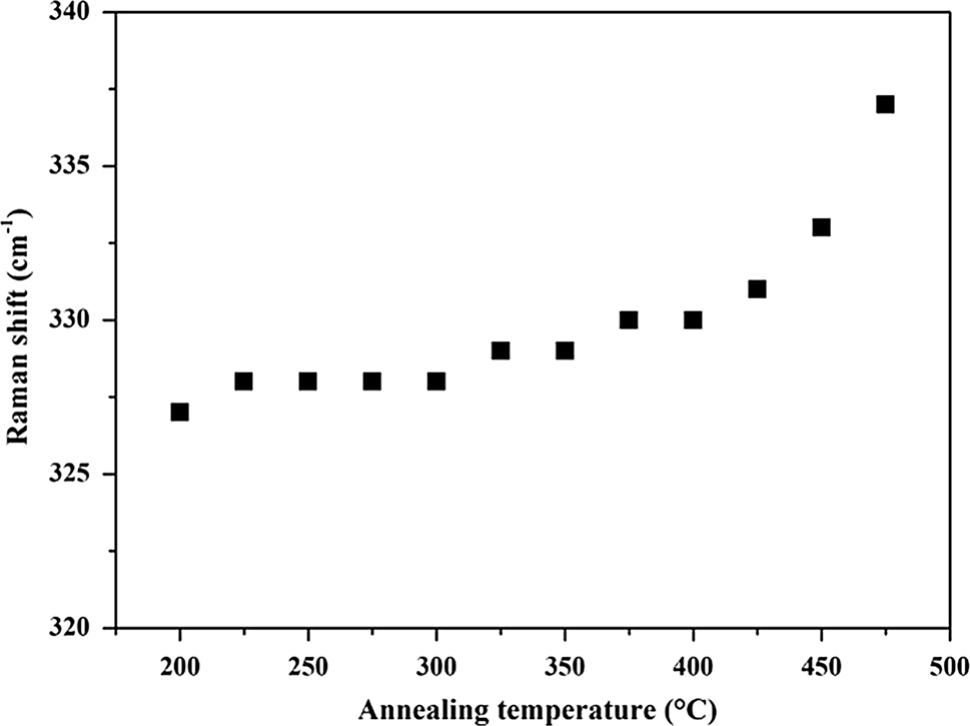
The position of the dominant Raman peak at 326–336 cm^−1^ in Fig. 3b and its relationship to the annealing temperature of the films

Figure 4 shows the dependence of the shift of the dominant Raman peak on the heating temperatures. The WZ-CZTS films have a major peak at 326–327 cm^−1^, and this can clearly be seen for the samples prepared at <350 °C. As the annealing temperature increases, the peak moves toward 336 cm^−1^, representing the presence of kesterite. This is demonstrated by the rapid increase in Raman shift seen from 375 to 475 °C. The p-XRD data show that the films prepared at 400–450 °C are KS-CZTS too, and the downshifted peak is due to disorder in Cu and Zn atoms in the sublattice. This is because the Cu and Zn atoms have a similar size and the difference between binding energy of the cations in the sublattice is small [65, 66].

**Figure 5:**
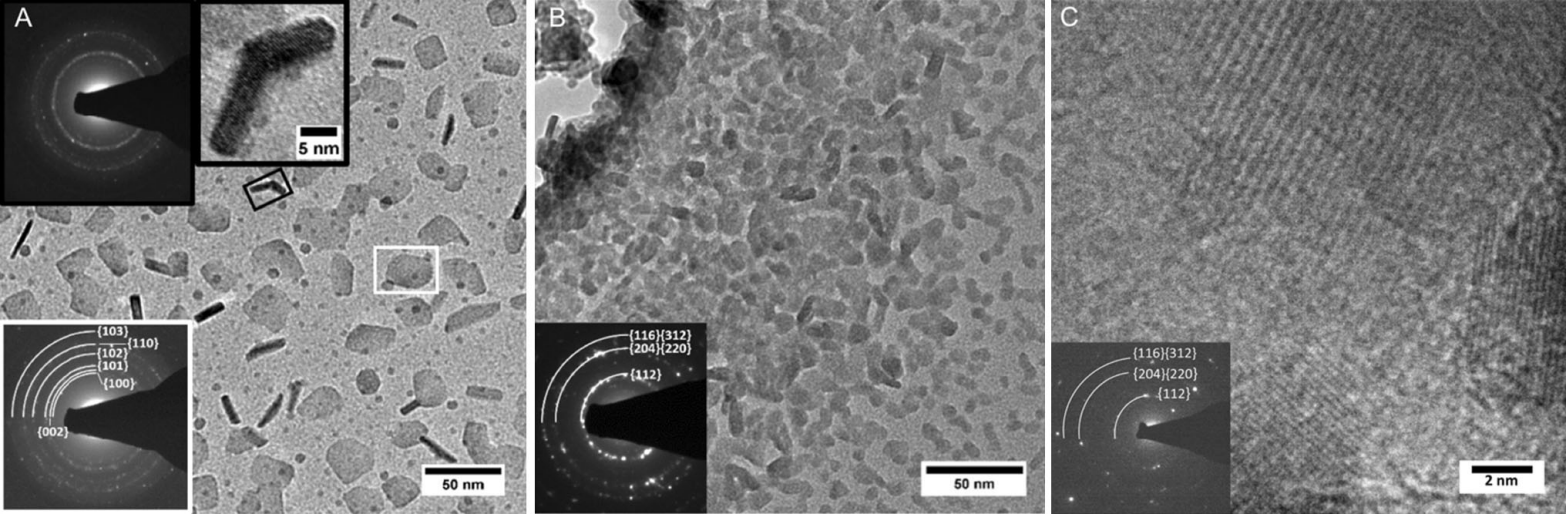
TEM images of Cu_2_ZnSnS_4_ nanocrystals. **a** A CZTS film heated at 225 °C. The insets show selected area electron diffraction (SAED) images, gray box for cubic CZTS and white box for hexagonal CZTS. **b** A film heated at 350 °C. The SAED pattern can be indexed to tetragonal CZTS. **c** A film heated at 450 °C, the inset SAED pattern is also indexed to tetragonal CZTS

Samples suitable for transmission electron microscope (TEM) imaging were prepared by ultrasonication of the CZTS film (produced at 225, 350 and 450 °C, Fig. 5). Closer inspection of the CZTS produced at 225 °C found that two distinct kinds of crystalline materials were present: a flake-like material and short nanorods with lattices fringes present (Fig. 5a). Both sets of crystals were typically <40 and 20 nm in diameter, respectively. We were able to observe patches on the TEM grid that consisted mainly of either phase. The selected area electron diffraction (SAED) patterns found distinct rings—indicative of polycrystalline materials, with these rings consistent with the identification of hexagonal (flake-like) and cubic (rod-like) phases of CZTS. This SAED, inset to Fig. 5a, shows an interplanar spacing of 0.27 and 0.19 nm, which correspond to the (200) and (220) planes of cubic CZTS (ESI Table S3). The interplanar spacing of 0.33 and 0.32 nm relates to the (100) and (002) planes of hexagonal CZTS (ESI Table S2). Two phases (hexagonal and cubic) can be seen in the p-XRD pattern of the film heated at 225 °C (Fig. 3a) due to the equivalence of the (002) planes of the hexagonal structure with the (111) planes of the cubic structure.

Exfoliation of CZTS films produced at 350 °C showed a crystalline material (<20 nm); SAED supported the formation of only the tetragonal phase of kesterite CZTS (Fig. 5b). The interplanar spacing of 0.31, 0.19 and 0.16 nm corresponds to the (112), {(204)(220)} and {(312)(116)} planes of kesterite CZTS (ESI Table S4). Likewise, for the film heated to 450 °C, we only observed crystalline particles <20 nm, and for which the SAED could also be indexed to tetragonal kesterite CZTS (Fig. 5c, ESI Table S5).

The electrical parameters of the WZ-CZTS film prepared at 225 °C (ESI S6) and the phase pure KS-CZTS samples annealed at 375 °C (ESI S7) and 450 °C (ESI S8) were obtained using the van der Pauw four-probe configuration in Hall effect measurements at room temperature. All samples exhibited p-type conductivity, a desirable requirement for the fabrication of heterojunction solar cells.

The resistivity (*ρ*), Hall mobility (*μ*), carrier concentration (*p*) and Hall coefficient (*R*_H_) are shown in Table 1. The resistivity (*ρ*) decreased from 27.1 Ω cm to around 1.23 Ω cm for the transition from WZ-CZTS to KS-CZTS. In addition, we found that the Hall coefficient (*R*_H_) decreased from 2.36 × 10^+3^ cm^3^ C^−1^ for WZ-CZTS films to 13.7 cm^3^ C^−1^ for KS-CZTS °C. We have determined that for the two phases, the Hall mobility (*μ*) is 87.1 and 11.1 cm^2^ V^−1^ s^−1^, respectively. The carrier concentration values were 2.65 × 10^+15^ and 4.55 × 10^+17^ cm^−3^, the latter of which is an excellent value for use in practical devices.

**Table 1:** Electrical properties of CZTS films prepared through melt reactions at 375 and 450 °C

T	225 °C	375 °C	450 °C
μ (cm^2^ v^−1^ s^−1^)	87.1	5.58	11.1
*p* (cm^−3^)	2.65 × 10^+15^	1.32 × 10^+18^	4.55 × 10^+17^
R_H_ (C^−1^ cm^3^)	2.36 × 10^+3^	4.73	13.7
ρ (Ω cm)	27.1	0.85	1.23
σ (S cm^−1^)	0.0369	1.18	0.81
R_S_ (Ω/□)	1.37 × 10^+5^	4.35 × 10^+3^	6.55 × 10^+3^
Conductivity	p-type	p-type	p-type

*T* annealing temperature, *μ* Hall mobility, *p* Hall carrier density, *R*_H_ Hall coefficient, *ρ* resistivity, *σ* conductivity and *R*_S_ sheet resistance

TEM images of the film prepared at 225 °C indicate that the film exhibited hexagonal CZTS as well as cubic (Fig. 5a). This confirms the presence of impurities in the predominantly wurtzite phase CZTS. McGill [67] and Hall [68] previously showed that as the impurity concentration increases, the mobility decreases. This behavior has also been seen for Si [69] and GaAs [70]. This model can be applied to the CZTS system, as moving from 225 to 375/450 °C we see a substantial decrease in mobility.

## Conclusions

Thermogravimetric analysis was used to investigate the decomposition of a series of *O*-*n*-butyl and *O*-ethylxanthate complexes of copper, tin and zinc. [(Ph_3_P)_2_CuS_2_COEt], [Zn(S_2_CO^n^Bu)_2_] and [Sn(S_2_COEt)_2_] were found to have compatible decomposition temperatures and were used for the formation of Cu_2_ZnSnS_4_ (CZTS) by spin coating followed by heating under N_2_. The p-XRD patterns of CZTS thin films were obtained after heating at temperatures between 200 and 475 °C and revealed a temperature dependence of the CZTS phase formed. Higher temperatures give the normal tetragonal phase CZTS, while low temperatures are mixed hexagonal and cubic phases. EDX measurements show that the Cu/(Zn + Sn) ratio was between 1 and 0.64. The measured resistivity, carrier concentration, mobility and Hall coefficient of films heated at 225, 375 and 450 °C, indicated fairly homogenous films. In this work the wurtzite CZTS has a much higher resistivity and mobility, but a much lower charge carrier density.

## Electronic supplementary material

Below is the link to the electronic supplementary material.

Supplementary material 1 (DOCX 196 kb)
