# Supplementary material for: The synthesis and characterization of Cu2ZnSnS4 thin films from melt reactions using xanthate precursors
Source: J Mater Sci. 2017 Jul 20;52(21):12761–71. doi: 10.1007/s10853-017-1367-0 (PMC6979526; doi:10.1007/s10853-017-1367-0)
Supplement: Supplementary file 1 — Supplementary material 1 (DOCX 196 kb) [file 10853_2017_1367_MOESM1_ESM.docx]

**Supporting information for: Synthesis and characterization of Cu_2_ZnSnS_4_ thin films from melt reactions using Xanthate precursors**

Mundher Al-Shakban,^a^ Peter D. Matthews,^b^ Nicky Savjani,^b^ Xiang L. Zhong,^a^ Yuekun Wang,^c^ Mohamed Missous^c^ and Paul O’Brien^a,b,*^

1. School of Materials, University of Manchester, Oxford Road, Manchester M13 9PL, UK. E-mail: paul.o’brien@manchester.ac.uk; Fax: +44 (0)161 275 4616; Tel: +44 (0)161 275 4653
2. School of Chemistry, University of Manchester, Oxford Road, Manchester, M13 9PL, UK.
3. School of Electrical and Electronic Engineering, University of Manchester, Oxford Road, Manchester M13 9PL, UK.

**Synthesis of precursors**

*1.1 Synthesis of bis(O-ethylxanthato)zinc(II)*

[Zn(S_2_COEt)_2_] was synthesized by adapting the literature procedure [1]. Potassium ethylxanthate (5.00 g, 0.031 mol) was dissolved in deionized water (50 ml), and ZnCl_2_ (1.81 g, 0.013 mol) was dissolved in a similar amount of water. The ZnCl_2_ solution was slowly added to the KS_2_COEt solution and stirred for 30 minutes leading to the formation of a white precipitate. The reaction mixture was then filtered and the white solid product dried to give [Zn(S_2_COEt)_2_] (3.45g, 0.011 mol, 85% yield). MPt: 128-132 °C.

Calc. for C_6_H_10_O_2_S_4_Zn (%): C 23.4, H 3.28, S 41.6, Zn 21.3; found: C 23.8, H 3.13, S 41.8, Zn 21.1.

FT-IR (cm^-1^): 2990 (w), 1867 (w). 1189 (s), 1122 (s), 1024 (s), 867.6 (w), 817.1 (w), 657.0 (w).

*1.2 Synthesis of tetrakis(O-ethylxanthato)tin(IV)*

[Sn(S_2_COEt)_4_] was prepared by a procedure that was modified from that described in literature [2]. SnCl_4_ (1.04 g, 0.0040 mol) was dissolved in toluene (50 ml) and added dropwise to a solution of potassium ethylxanthate (2.80 g, 0.017 mol) in toluene (50 ml) and stirred for 1 h at room temperature. After filtering, the toluene solution was evaporated under reduced pressure and the oily residue shaken with 50 ml hexane and left to crystallize to give yellow crystals of [Sn(S_2_COEt)_4_] (1.80 g, 0.0030 mol, 75% yield). MPt: 58-62 °C.

Calc. for C_12_H_20_O_4_S_8_Sn (%): C 23.9, H 3.34, S 42.4, Sn 19.7; found: C 24.4, H 3.36, S 42.1, Sn 20.1.

FT-IR (cm^-1^): 2983 (w), 2932 (w), 1459 (w), 1365 (w), 1233 (s), 1142(m), 1020 (s), 848.1 (w), 807.8 (w), 566.8 (w).

*1.3 Synthesis of (O-butylxanthato)copper(I) triphenylphosphine*

A solution of potassium butylxanthate (0.75 g, 0.0040 mol) in chloroform (40 ml) was added to a solution of triphenylphosphine (2.09 g, 0.008 mol) and CuCl (0.40 g, 0.0040 mol) in the same amount of chloroform. A white precipitate was obtained after continuous stirring for 1 h at room temperature. The solution was filtered to obtain a clear yellow solution. Cooling the yellow solution to -20 °C gave yellow crystals of O-butylxanthato copper(I) triphenyl-phosphine (2.10 g, 0.0028 mol, 71% yield). MPt: 132-137 °C.

Calc. for C_41_H_39_CuOP_2_S_2_ (%): C 66.8, H 5.33, S 8.67, P 8.40, Cu 8.62; found: C 66.3, H 5.46, S 8.00, P 8.64, Cu 8.93.

FT-IR (cm^-1^): 3061 (w), 2998 (w), 1478 (m). 1433 (m), 1313 (s), 1168 (m), 1092 (m), 1052 (s), 996.1 (s), 743.6 (m), 618.5 (s), 574.1 (s).

**Table S1.** Composition percentages and ratios for CZTS films after annealing at different temperatures as determined by EDX.

| **T (**°**C )** | **Cu** | **Zn** | **Sn** | **S** | **Zn/Sn** | **Cu/(Zn+Sn)** |
| --- | --- | --- | --- | --- | --- | --- |
| **200** | 28 | 17 | 14 | 41 | 1.2 | 0.9 |
| **225** | 30 | 13 | 17 | 40 | 0.8 | 1.0 |
| **250** | 29 | 17 | 14 | 41 | 1.2 | 1.0 |
| **275** | 28 | 18 | 16 | 38 | 1.1 | 0.8 |
| **300** | 30 | 18 | 13 | 39 | 1.3 | 1.0 |
| **325** | 28 | 15 | 16 | 41 | 1.0 | 0.9 |
| **350** | 28 | 18 | 14 | 39 | 1.3 | 0.9 |
| **375** | 29 | 16 | 15 | 40 | 1.1 | 0.9 |
| **400** | 27 | 18 | 15 | 39 | 1.2 | 0.8 |
| **425** | 28 | 19 | 15 | 38 | 1.3 | 0.8 |
| **450** | 28 | 17 | 16 | 39 | 1.1 | 0.8 |
| **475** | 28 | 18 | 14 | 39 | 1.3 | 0.9 |

**Figure S1.** The elemental composition of the films determined by EDX.

**Figure S2.** Elemental composition of the CZTS films determined by EDX normalised to Cu_2_.

**Table S2.** Lattice parameters calculated from p-XRD and SAED (Figure 5a) for hexagonal CZTS film heated at 225 °C.

| d [Å] from TEM | hkl | Calc. unit cell from TEM [Å] | | | Calc. unit cell from PXRD [Å] | | |
| --- | --- | --- | --- | --- | --- | --- | --- |
|  |  | a | b | c | a | b | c |
| 3.3 | (100) | 3.80 | 3.80 | 6.32 | 3.83 | 3.83 | 6.30 |
| 3.2 | (002) |  |  |  |  |  |  |
| 2.9 | (101) |  |  |  |  |  |  |
| 6.3 | (102) |  |  |  |  |  |  |
| 1.9 | (110) |  |  |  |  |  |  |
| 1.7 | (103) |  |  |  |  |  |  |

**Table S3.** Lattice parameters calculated from p-XRD and SAED (Figure 5a) for cubic CZTS heated at 225 °C.

| d [Å] | hkl | Calc. unit cell from TEM [Å] | | | Calc. unit cell from PXRD [Å] | | |
| --- | --- | --- | --- | --- | --- | --- | --- |
|  |  | a | b | c | a | b | c |
| 2.70 | (200) | 5.42 | 5.42 | 5.42 | 5.43 | 5.43 | 5.43 |
| 1.91 | (220) |  |  |  |  |  |  |

**Table S4**. Lattice parameters calculated from p-XRD and SAED for tetragonal CZTS prepared at 350 °C (Figure 5b).

| d [Å] | hkl | Calc. unit cell from TEM [Å] | | | Calc. unit cell from PXRD [Å] | | |
| --- | --- | --- | --- | --- | --- | --- | --- |
|  |  | a | b | c | a | b | c |
| 3.13 | (112) | 5.43 | 5.43 | 10.84 | 5.43 | 5.43 | 10.85 |
| 1.91 | (204)(220) |  |  |  |  |  |  |
| 1.63 | (116)(312) |  |  |  |  |  |  |

**Table S5**. Lattice parameters calculated from p-XRD and SAED for tetragonal CZTS prepared at 450 °C (Figure 5c).

| d [Å] | hkl | Calc. unit cell from TEM [Å] | | | Calc. unit cell from PXRD [Å] | | |
| --- | --- | --- | --- | --- | --- | --- | --- |
|  |  | a | b | c | a | b | c |
| 3.13 | (112) | 5.43 | 5.43 | 10.80 | 5.43 | 5.43 | 10.84 |
| 1.91 | (204)(220) |  |  |  |  |  |  |
| 1.59 | (116)(312) |  |  |  |  |  |  |

**Table S6.** Lattice parameters calculated from p-XRD for tetragonal and hexagonal CZTS prepared by heating spin-coated films at various temperatures. The literature values of lattice parameters are a = b = 5.43 Å and c = 10.84 Å for tetragonal [3] and a = b = 3.83 Å, c = 6.31 Å for hexagonal [4].

| T (^o^C) | Calc. unit cell for tetragonal CZTS [Å] | | | Calc. unit cell for hexagonal CZTS [Å] | | |
| --- | --- | --- | --- | --- | --- | --- |
|  | a | b | c | a | b | c |
| 200 |  |  |  | 3.837 | 3.837 | 6.300 |
| 225 |  |  |  | 3.832 | 3.832 | 6.301 |
| 250 |  |  |  | 3.836 | 3.836 | 6.299 |
| 275 |  |  |  | 3.837 | 3.837 | 6.302 |
| 300 |  |  |  | 3.836 | 3.836 | 6.299 |
| 325 |  |  |  | 3.837 | 3.837 | 6.302 |
| 350 |  |  |  | 3.831 | 3.831 | 6.297 |
| 375 | 5.433 | 5.433 | 10.844 |  |  |  |
| 400 | 5.429 | 5.429 | 10.845 |  |  |  |
| 425 | 5.431 | 5.431 | 10.844 |  |  |  |
| 450 | 5.431 | 5.431 | 10.844 |  |  |  |
| 475 | 5.431 | 5.431 | 10.844 |  |  |  |

**
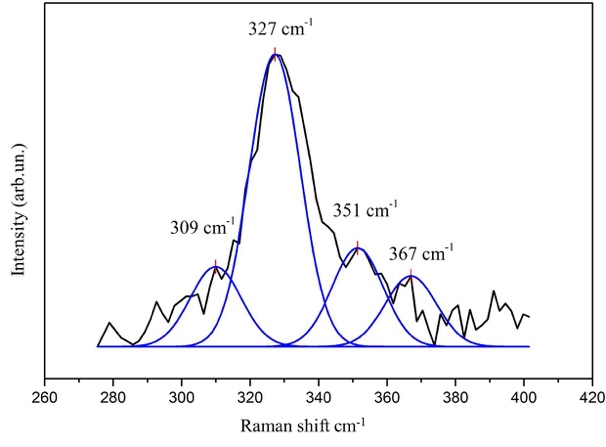
**

**Figure S3.** Raman spectrum of film prepared at 200 °C.

**
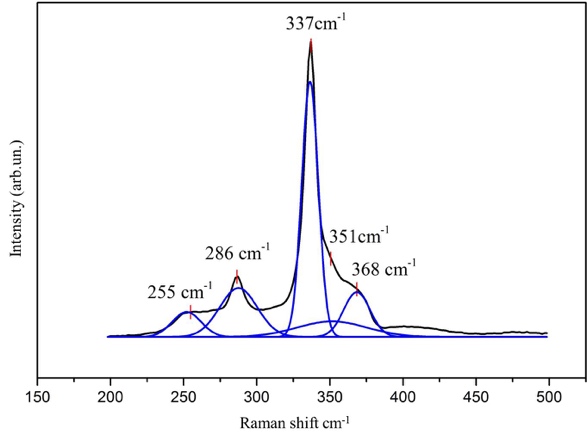
**

**Figure S4.** Raman spectrum of film prepared at 475 °C.

**S7. Electrical measurements for the sample prepared at 225 °C**

--------------------------------------------------------------------

Resistivity measurement (I = current, V = voltage, [XY] = measurement between points X and Y):

| I[21] = 0.012 mA | V[43] = 463.400 mV |
| --- | --- |
| I[12] = 0.012 mA | V[43] = -618.100 mV |
| I[32] = 0.012 mA | V[41] = 891.100 mV |
| I[23] = 0.013 mA | V[41] = -938.200 mV |
| I[43] = 0.012 mA | V[12] = 497.500 mV |
| I[34] = 0.012 mA | V[12] = -569.600 mV |
| I[14] = 0.012 mA | V[23] = 829.000 mV |
| I[41] = 0.012 mA | V[23] = -949.600 mV |

Hall measurement without magnetic field (I = current, V = voltage, [XY] = measurement between points X and Y):

| I[31] = 0.012 mA | V[42] = 341.500 mV |
| --- | --- |
| I[13] = 0.012 mA | V[42] = -400.400 mV |
| I[42] = 0.012 mA | V[13] = -412.700 mV |
| I[24] = 0.012 mA | V[13] = 322.300 mV |

Hall measurement with applied 0.088 T magnetic field (I = current, V = voltage, [XY] = measurement between points X and Y):

| I[31] = 0.012 mA | V[42] = 344.200 mV |
| --- | --- |
| I[13] = 0.012 mA | V[42] = -403.100 mV |
| I[42] = 0.012 mA | V[13] = -415.300 mV |
| I[24] = 0.012 mA | V[13] = 324.300 mV |

Hall Voltages:

Hall Voltage 1 = 2.700 mV

Hall Voltage 2 = 2.700 mV

Hall Voltage 3 = 2.600 mV

Hall Voltage 4 = 2.000 mV

**S8. Electrical measurements results for sample prepared at 375 °C**

----------------------------------------------------------------

Resistivity measurement (I = current, V = voltage, [XY] = measurement between points X and Y):

| I[21] = 1.202 mA | V[43] = 2672.400 mV |
| --- | --- |
| I[12] = 1.205 mA | V[43] = -2674.300 mV |
| I[32] = 1.201 mA | V[41] = 1817.900 mV |
| I[23] = 1.204 mA | V[41] = -1818.600 mV |
| I[43] = 1.201 mA | V[12] = 2674.400 mV |
| I[34] = 1.205 mA | V[12] = -2674.600 mV |
| I[14] = 1.201 mA | V[23] = 1818.200 mV |
| I[41] = 1.204 mA | V[23] = -1819.300 mV |

Hall measurement without magnetic field (I = current, V = voltage, [XY] = measurement between points X and Y):

| I[31] = 1.201 mA | V[42] = -859.100 mV |
| --- | --- |
| I[13] = 1.202 mA | V[42] = 856.400 mV |
| I[42] = 1.203 mA | V[13] = 853.600 mV |
| I[24] = 1.203 mA | V[13] = -855.000 mV |

Hall measurement with applied 0.088 T magnetic field (I = current, V = voltage, [XY] = measurement between points X and Y):

| I[31] = 1.202 mA | V[42] = -858.100 mV |
| --- | --- |
| I[13] = 1.202 mA | V[42] = 856.000 mV |
| I[42] = 1.203 mA | V[13] = 853.300 mV |
| I[24] = 1.203 mA | V[13] = -854.700 mV |

Hall Voltages:

Hall Voltage 1 = 1.000 mV

Hall Voltage 2 = 0.400 mV

Hall Voltage 3 = 0.300 mV

Hall Voltage 4 = 0.300 mV

**S9. Electrical measurements results for the sample prepared at 450 °C**

--------------------------------------------------------------------

Resistivity measurement (I = current, V = voltage, [XY] = measurement between points X and Y):

| I[21] = 0.112 mA | V[43] = 214.350 mV |
| --- | --- |
| I[12] = 0.112 mA | V[43] = -240.240 mV |
| I[32] = 0.112 mA | V[41] = 379.700 mV |
| I[23] = 0.112 mA | V[41] = -381.900 mV |
| I[43] = 0.112 mA | V[12] = 227.080 mV |
| I[34] = 0.112 mA | V[12] = -228.920 mV |
| I[14] = 0.112 mA | V[23] = 376.600 mV |
| I[41] = 0.112 mA | V[23] = -381.700 mV |

Hall measurement without magnetic field (I = current, V = voltage, [XY] = measurement between points X and Y):

| I[31] = 0.112 mA | V[42] = 151.680 mV |
| --- | --- |
| I[13] = 0.112 mA | V[42] = -154.050 mV |
| I[42] = 0.112 mA | V[13] = -153.120 mV |
| I[24] = 0.112 mA | V[13] = 150.250 mV |

Hall measurement with applied 0.088 T magnetic field (I = current, V = voltage, [XY] = measurement between points X and Y):

| I[31] = 0.112 mA | V[42] = 151.770 mV |
| --- | --- |
| I[13] = 0.112 mA | V[42] = -154.210 mV |
| I[42] = 0.112 mA | V[13] = -153.280 mV |
| I[24] = 0.112 mA | V[13] = 150.380 mV |

Hall Voltages:

Hall Voltage 1 = 0.090 mV

Hall Voltage 2 = 0.160 mV

Hall Voltage 3 = 0.160 mV

Hall Voltage 4 = 0.130 mV

**SEM Images**

**
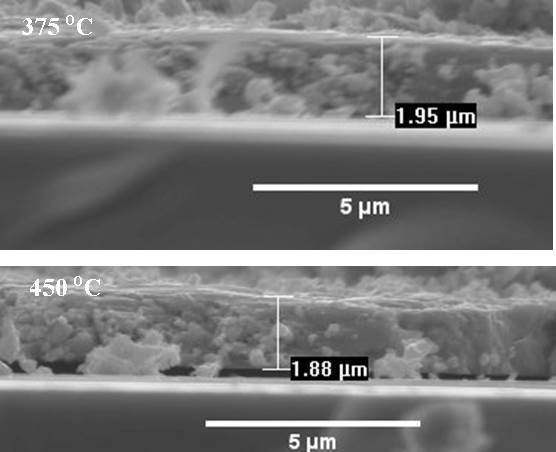
**

**Figure S5.** Side-on SEM image of a film prepared at 375 °C and 450 °C.

**Film Image**

**
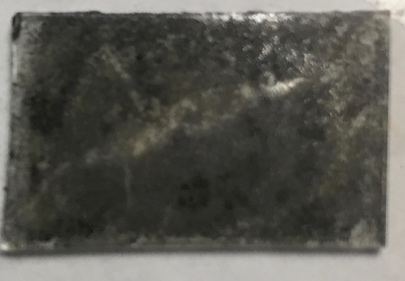
**

**Figure S6.** Example image of the films prepared by spin-coating and annealing. This example was prepared at 350 °C.

**References**

[1] T. Ikeda, H. Hagihara, The crystal structure of zinc ethylxanthate, Acta Crystallogr. 21 (1966) 919–927. doi:10.1107/S0365110X66003529.

[2] C.L. Raston, P.R. Tennant, A.H. White, G. Winter, Reactions of Tin(II) and Tin(IV) Xanthates: Crystal Structure of Tetrakis (O-ethylxanthato) tin(IV), Aust. J. Chem. 31 (1978) 1493–1500. doi:10.1071/CH9781493.

[3] P. Bonazzi, L. Bindi, G.P. Bernardini, S. Menchetti, A Model for the Mechanism of Incorporation of Cu, Fe, and Zn in the Stannite-Kesterite Series, Cu2FeSnS4 - Cu2ZnSnS4, Can. Mineral. 41 (2003) 639–647. doi:10.2113/gscanmin.41.3.639.

[4] M. Li, W. Zhou, J. Guo, Y. Zhou, Z. Hou, J. Jiao, et al., Synthesis of Pure Metastable Wurtzite CZTS Nanocrystals by Facile One-Pot Method, J. Phys. Chem. C. 116 (2012) 26507–26516. doi:10.1021/jp307346k.
